# A spontaneous splenic rupture secondary to Epstein Barr Virus infection: a case report

**DOI:** 10.1016/j.ijscr.2025.111609

**Published:** 2025-07-02

**Authors:** Giulia Munzi, James Rossario Casella Mariolo, Gaetano Poillucci, Alessandra Iodice, Laura Rossi, Angelo Serao

**Affiliations:** Department of General and Emergency Surgery, Ospedale dei Castelli (NOC), ASL Roma 6, Rome, Italy

**Keywords:** Spleen, Atraumatic splenic rupture, Epstein Barr Virus, Mononucleosis, Case report

## Abstract

**Introduction:**

Atraumatic Splenic Rupture (ASR) is a very rare and potentially life-threatening event associated with Epstein Barr Virus (EBV)-induced mononucleosis; it occurs in <0.5% of overall cases. The aim of the present study was to publish a case report of the ASR and to present a mini review of the international literature.

**Case presentation:**

A 22-year-old female presented to emergency department with abdominal pain, nausea and vomiting. The patient's medical history was unremarkable, and she denied trauma. On arrival, her vital signs were stable. However, laboratory examinations showed haemoglobin values of 7.8 g/dl, lymphocytosis and elevated liver function tests. Abdomen Computed Tomography (CT) scan highlighted haemoperitoneum and splenic hematoma. Angiography and embolization of active bleeding followed by emergency laparoscopic splenectomy were performed.

**Discussion:**

In patients without a history of trauma, spontaneous splenic rupture should be considered in the differential diagnosis if patients have abdominal and left shoulder tip pain, and laboratory results indicate low haemoglobin level. There is no consensus on treatment management on ASR.

**Conclusion:**

This case underlines the importance of considering splenic rupture as a complication of EBV infection. Moreover, endovascular techniques and laparoscopic management can be considered to ensure a minimally invasive approach and improve outcomes.

## Highlights

• ASR is a very rare and potentially life-threatening event;
• ASR associated can be a complication of EBV infection;
• Endovascular techniques and laparoscopic management can be considering to ensure a minimally invasive approach and improve outcomes.

## 1 Introduction

The most common cause of damage to the spleen is the abdominal trauma; 93% of patients with atraumatic splenic rupture are attributed to pathological causes, whereas idiopathic causes account for 7% of cases [1]. Infectious Mononucleosis (IM) is a contagious illness induced by the Epstein Barr Virus (EBV). IM clinical presentation may be variable but it is generally a self-limiting condition. Usually, patients with IM present with a clinical picture of the triad pharyngitis, cervical lymphadenopathy, and fever [2]. Hepatomegaly and splenomegaly may be present [3].

Atraumatic Splenic Rupture (SR) is a very rare but potentially lethal complication of IM occurring in 0.1% 0.5% percent of cases [4] and 86% of those occurring atraumatically [5].

The cardinal symptoms of SR include left upper quadrant pain or tenderness, left shoulder tip pain (Kehr sign), abdominal distension, syncope, and hypotension. Abdominal UltraSonography (US) and Computed Tomography (CT) is suitable for haemodynamically stable patients and provides assessment of all abdominal organs [6].

Management of SR can be divided into non operative management in hemodinamically stable patients and operative management in unstable patients [7].

There are three methods of operative management of SR [8]: open splenectomy, laparoscopic splenectomy, combination of pre-operative splenic artery embolization and laparoscopic splenectomy. Despite the minimally invasive approach, decreased intraoperative blood loss when compared with other methods, there are few cases reported in the literature.

The purpose of the present paper was to present the complication of atraumatic SR in IM and to highlight the importance of diagnosing EBV-associated SR and the successful minimally invasive management to improve outcome.

Moreover, PubMed, Embase, Google Scholar, Medline and Cochrane Library systematically searched to write a mini-review.

After these considerations, we proceed to discuss a rare case of atraumatic SR secondary to EBV infection in a 22-year-old female, underscoring the challenges in management according to the SCARE checklist [9].

## 2 Case presentation

A 22-year-old female presented to emergency department with abdominal pain, nausea and vomiting following syncopal episode. She denied any trauma. The patient reported fever, myalgia and arthralgia for 10 days. The patient's medical history was unremarkable; she has not undergone any surgical procedures. Her past medical and family history was unremarkable. She did not report any allergies.

At hospital admission the patient was hemodynamically stable: she had a blood pressure of 130/70 mmHg, heart rate of 98 bpm, oxygen saturation 98 %, no fever and a Glasgow Coma Scale of 15.

Physical examination revealed diffuse abdominal pain with tenderness in the left upper quadrant, left shoulder tip pain, prolonged capillary refill time, and neck lymphadenopathy.

Laboratory results were: haemoglobin 7.8 g/dl, white blood cell count 11.000/mm^3^ (neutrophils 21%, lymphocytes 69%), C-reactive protein 10.2 mg/dl, aspartate aminotransferase 198 U/l, alanine aminotransferase 220 U/l. Serology of EBV was conducted in suspicion of IM.

**Fig. 1:**
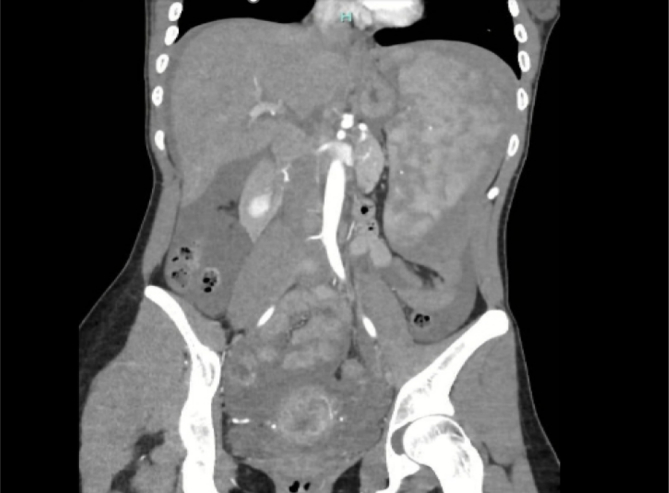
Abdominal CT-scan (coronal view).

**Fig. 2:**
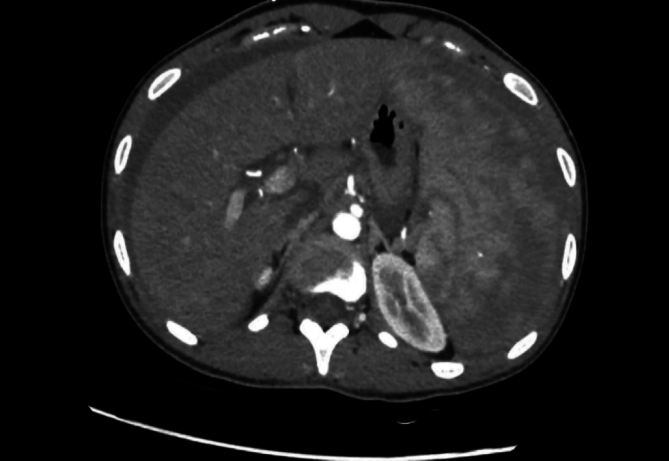
Abdominal CT-scan (axial view).

Abdominal CT-scan (Fig. 1, Fig. 2) revealed the presence of haemoperitoneum, splenomegaly (20 cm in diameter) and a splenic hematoma with signs of active bleeding (AAST splenic injury grade III [7]).

**Fig. 3:**
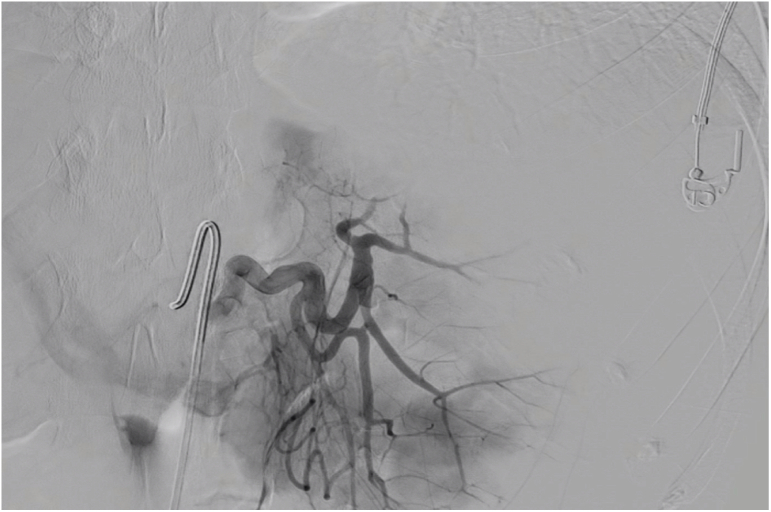
Angiography and embolization of active bleeding.

The patient maintained hemodynamic stability, and an angiography was performed: embolization of the source of active bleeding of the upper pole of the spleen by Polyvinyl Alcohol particles was performed (Fig. 3).

**Fig. 4:**
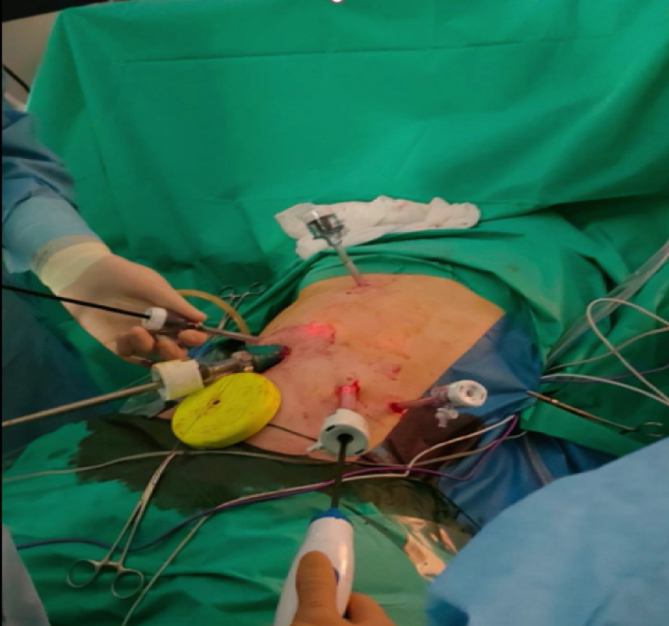
Trocars placement.

**Fig. 5:**
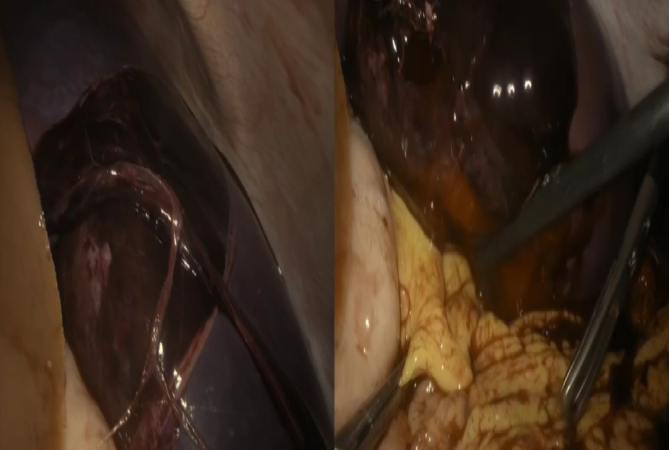
Splenic hematoma.

Subsequently, the patient underwent an emergency laparoscopic exploration: haemoperitoneum, splenomegaly with haematoma identified on the ruptured capsule with lots of blood clots were confirmed; it was performed a laparoscopic splenectomy and an abdominal drain was inserted at the end of the procedure (Fig. 4, Fig. 5).

Patient's postoperative course was regular with progressive recovery of intestinal motility and stabilization of vital signs without additional transfusion needs. She received Pneumococcal, Haemophilus Influenza and Meningococcal vaccines on 3rd postoperative day. Infection disease serology was positive for the presence of EBV VCA IgM. The patient was discharged on the seventh postoperative day.

## 3 Discussion

IM caused by EBV is usually self-limited disease and it resolves over a period of weeks or months without sequelae. Although, complications have been recognised: periorbital and/or palpebral edema, splenomegaly, hepatomegaly, skin rash and chronic fatigue syndrome [2]. In this case, the diagnosis was supported by clinical presentation to emergency department, lymphocytosis, and the deranged liver function tests.

Atraumatic SR are very uncommon with an incidence rate of 3.2% [10]; when occur, it has a mortality rate of 12% 20% [11]. It is defined by these criteria: detailed history not revealing any history of trauma, no evidence of disease in any organs apart from the spleen, no evidence of perisplenic adhesions or scarring consistent with previous trauma history, and a macroscopic and histological examination that shows the spleen to be normal [12]. In 1991, Crate and Payne [13] added a fifth criterion: they stated that no increase inantibodies, suggestive of viral infection in the acute or convalescent phase, should be confirmed, and they considered viraemia as a cause of rupture.

The pathological causes of atraumatic SR can be divided into seven categories: infectious, haematological, neoplastic, inflammatory, iatrogenic, primary splenic, and idiopathic; infectious diseases and neoplasia account for more than half of all cases [14,15].

Liu et al. [10] suggested the use of the term atraumatic because is more accurate and the conditions can be classified according to etiological factors and pathological changes as “atraumatic-pathological SR” and “atraumatic-idiopathic SR”.

There are no confirmed risk factors for atraumatic SR. In a study by Kocael et al. [11], they demonstrated that the most common etiological factor was a history of anticoagulant/antiaggregant drug use (33%).

Splenomegaly occurs in approximately 50% in the context of IM [2]. Spleen enlargement caused by lymphocytic infiltration leads to a greater susceptibility to rupture, which is generally atraumatic [16]. Atraumatic SR is an uncommon complication of IM, but it is the most common cause of death in these cases, with a mortality of 9% [5].

A systematic review by Bartlett et al. [5] of case reports of SR in IM between 1984 and 2014 outlines the more commonly signs: abdominal pain (88%), Kehr sign (33%), collapse or circulatory shock (27%); approximately 80% of cases occurred within three weeks of the onset of mononucleosis symptoms. NICE guidance [17] recommended to avoid contact sports for one month onset of symptoms to reduce the risk of SR.

To diagnose traumatic splenic injuries, contrast enhanced CT has nearly 100% accuracy, while the sensitivity of contrast enhanced CT in diagnosing atraumatic SR is 85.7% [10]. The sensitivity of US for diagnosing atraumatic SR is 57.1 % and, consequentially, CT is the imaging modality of choice for patients with haemodynamic stability [6].

The American Association for the Surgery of Trauma (AAST) splenic injury scale is the most widely used grading system for splenic trauma; it considered the anatomical lesions [7]. In our case, CT scan demonstrated a AAST grade III injury due to the presence of a subcapsular haematoma, involving >50% of the surface area.

There is no clear consensus on treatment strategy in patient with SR in IM as this occurs as a rare complication [18]. Treatment strategy decisions were based on haemodynamic status, CT grading, and etiological factors. In traumatic SR, hemodinamically stable patient undergo to non operative management according to World Society of Emergency Surgery (WSES) guidelines [7]; spleen preserving treatment is an alternative to splenectomy to reduce immunodeficiency following surgical spleen removal. However, the risk of potential death from SR in IM overcomes the risks of sepsis from post-splenectomy infection. However, a high failure rate has been reported [19].

The management of SR in IM was described in 82 cases by Barlett et al. [5]: splenectomy was performed in 67% of patients, non operative management was performed in 28% of cases, 5% had an initial trial of non operative management that failed necessitating splenectomy.

In a recent systematic review by Toti et al. [18] were described 144 cases of SR in IM categorised as minor, moderate and severe in according with the scoring system reccomended by WSES for patient with splenic trauma [7]. Immediate splenectomy (*n*=108) or after failure of non-operative management (*n*=15) was the most common treatment strategy. In the remaining 63 patients, the management was non-operative.

A literature review by Wu et al. [8] compared three methods of surgery to treat splenomegaly in a series of 79 patients: an open splenectomy was performed in 20 patients, a laparoscopic splenectomy in 30 patients, and a combination of preoperative splenic artery embolization and laparoscopic splenectomy in 29 patients. This study suggested that patients undergoing angiography and laparoscopic splenectomy had the potential to reduce operating time, intraoperative blood loss, and shorter postoperative hospital stay.

According to WSES guideline hemodynamic stability allowed to perform angiography/embolization [7]; subsequently a laparoscopic splenectomy was performed thus to reduce the risk of potential death from SR in IM. Combination of these minimally invasive approch allowed to improve outcomes: stabilization of vital signs without additional transfusion needs during hospitalization, patient's discharged on the seventh postoperative day.

Splenectomy is the surgery of choice and it is justified for three reasons: splenectomy helps identify underlying pathologies, the immunological function of the spleen may have been lost, the spleen is oedematous and fragile [10].

## 4 Conclusion

Clinicians need to be aware of the variable of SR as a complication of IM. There is the risk of ongoing bleeding and potential for late rupture due to splenomegaly. The treatment strategy should be chosen with caution. This case report underlines the feasible of a minivasive approach to improve outcome: angiography with embolization primarily stabilized the patient to facilitate a safer laparoscopic procedure.

## Informed consent

Written informed consent was obtained from the patient for publication and any accompanying images. A copy of the written consent is available for review by the Editor-in-Chief of this journal on request.

## Consent for publication

All Authors provide consent for publication.

## Ethical approval

This case report does not require ethical approval as it involves a single patient case that is anonymized and does not include any identifiable personal information. The patient provided informed consent for the publication of this report.

## Guarantor

The Guarantor of this case report is Gaetano Poillucci.

## Funding

There are no funding sources.

## Author contribution

Giulia Munzi study conception and design, literature search, data acquisition, interpretation and analysis; final approval of the version to be published. James Rossario Casella Mariolo drafting and critically revising the article for important intellectual content; final approval of the version to be published. Gaetano Poillucci drafting and critically revising the article for important intellectual content; final approval of the version to be published. Alessandra Iodice study conception and design, literature search, data acquisition, interpretation and analysis; final approval of the version to be published. Laura Rossi literature search, data acquisition, interpretation and analysis; final approval of the version to be published. Angelo Serao drafting and critically revising the article for important intellectual content; final approval of the version to be published.

## Declaration of competing interest

The Authors declare that they have no competing interests.
